# Drinking water services in the primary schools: evidence from coastal areas in Bangladesh

**DOI:** 10.1016/j.heliyon.2022.e09786

**Published:** 2022-06-23

**Authors:** Mohammad Jobayer Hossain, Md. Ansarul Islam, Md. Hasibur Rahaman, Md. Arif Chowdhury, Md. Atikul Islam, Mohammad Mahfuzur Rahman

**Affiliations:** a Department of Environmental Science and Technology, Jashore University of Science and Technology, 7408, Jashore, Bangladesh; b Department of Climate and Disaster Management, Jashore University of Science and Technology, 7408, Jashore, Bangladesh; c Environmental Science Discipline, Khulna University, 9208, Khulna, Bangladesh

**Keywords:** Coastal Bangladesh, Drinking water, Primary school, Water quality, Water supply

## Abstract

Salinity intrusion both in surface and groundwater caused a crisis for safe drinking water in coastal Bangladesh. The situation is even worse for children especially at school. However, information on water services in coastal schools is limited. Here we assess the quality of drinking water and supply infrastructures in the primary schools of a severely saline affected coastal area of Bangladesh. To fulfill the objective, thirty-eight schools were purposively selected and investigated in Dacope Upazila of Khulna district in Bangladesh. Findings revealed that harvested rainwater (63%) and pond (21%) are the major drinking water sources where countries’ leading water supply technology, tube well (16%) were the least used option. Moreover, salinity in all the tube wells exceeded the national standard. DO, pH, NO_3_, SO_4_ and PO_4_ concentration of all options satisfied national standards. However, total coliform counts exceeded the national standard. More than half of the samples had a low to high risk of indicator bacteria which is a major public health concern. Although 29% schools have installed portable water filtration units, those are grossly inaccessible for the students. Hence, students are reportedly consuming unsafe drinking water, and thus are vulnerable to water-borne diseases. The lack of resources and poorly designed infrastructure are the principal challenges to the safe drinking water supply. Therefore, disinfection at the point of use along with proper maintenance of the water infrastructure is urgent needs to safeguard potable water services in the primary schools of coastal Bangladesh.

## Highlights

• 38 primary schools were evaluated for drinking water quality covering all sources.
• Microbial contamination at both sources and point of use was identified.
• 1 in 5 school depends heavily on the contaminated surface water sources.
• 3 in 10 schools had water filtration facilities, primarily for the teachers.
• About half of the sources can be identified as highly risky based on *E. coli* count.

## Graphical abstract

**Figure:**
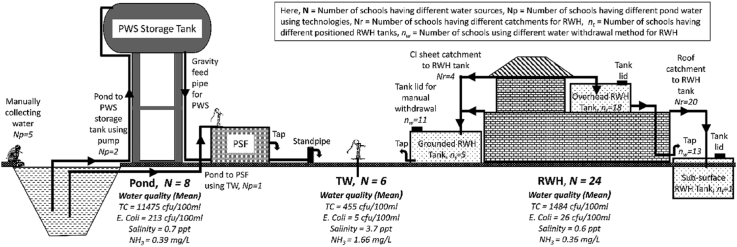

## 1 Introduction

Access to safe drinking water has been rated as the most essential element for sustaining human life. Every year millions of people suffer from diarrhea, cholera, typhoid, and parasites due to the consumption of unsafe water. Bangladesh has been relied heavily on groundwater for safe drinking water (WHO/UNICEF, 2018). However, the provision of drinking water supply system in the coastal areas of Bangladesh is very complex due to saline water intrusion (Rakib et al., 2020; Zahid et al., 2018). Moreover, salinity levels in surface and sub-surface waters have increased in manifold during the last three decades (Hasan et al., 2020; Zahid et al., 2018). Therefore, availability of freshwater aquifers in a feasible depth are scarce in these regions (Hoque et al., 2019).

People living in coastal Bangladesh adopted diverse techniques, varies temporally and spatially, to meet their drinking water requirements (Hoque et al., 2019; Islam et al., 2013). In Dacope Upazila (one of the salinity affected coastal sub-district), people principally dependents on rainwater for their drinking water requirements, through rain fed-pond and rainwater harvesting (RWH) in storage tanks (Islam et al., 2013). People used to preserve rainwater during the rainy season and store it for uses during non-rainy times of the year. In a typical RWH system, the storage tanks receive feed-water directly from the roof catchment without any pretreatment (Islam et al., 2013, 2019). In general, rainwater is comparatively safe but not free from contaminations. The cleanliness and management efforts in the rainwater capturing and storing process determines the chances of contamination and the quality of the harvested water (Alim et al., 2020). Residents are often ignorant about the non-visual contamination or cannot afford proper maintenance due to the financial constraints or technical incapability. Therefore, the presence of pathogens becomes the most common incidence of contamination. Waterborne diseases are widespread within the coastal communities and could have a disastrous impact on the public health and nutrition, particularly on school-aged children (Ding et al., 2017).

Previous studies extensively reported the effect of drinking water salinity and microbial contamination on the public health of the coastal regions. For instance, Khan et al. (2011) established a link between drinking water salinity and the prevalence of hypertension in pregnant women. Also, Dasgupta et al. (2011) documented drinking water salinity as one of the determinants of infant mortality in coastal Bangladesh. Numerous studies also reported salinity and bacterial contamination with higher incidences of water-borne illnesses including diarrhea, dysentery, cholera, jaundice in coastal Bangladesh (Asma and Kotani, 2021; Hossain et al., 2021; Rahman et al., 2017; Islam et al., 2011). More recently, Akter (2019) argued that drinking water salinity decreases the children’s grade advancement likelihood. Moreover, UNICEF (2006) has reported that school-going children in coastal areas of Bangladesh are mostly vulnerable to different forms of water-borne diseases. The ages of primary school-going children in Bangladesh typically range between 4 and 12 years. Students of grades 1 to 5 used to spend most of the day time in their schools (Kaiser et al., 2001). Students carrying their personal drinking water with them is very common in the cities. In contrast, students from the countryside, particularly in the coastal areas are not accustomed with carrying water from their home or simply cannot afford to avail it. Therefore, they must rely on the water available at their schools. However, the water supply aspect in the schools did not get much attention in Bangladesh. Decades of less prioritization or negligence allowed propagation of challenges to ensure safe drinking water for the children (World Bank, 2018).

In Bangladesh, only 73% of the primary schools were estimated to have basic water services (WHO/UNICEF, 2018). However, the services are not equal all over the country, highly varies spatially, particularly inferior in coastal areas. Despite the importance, the scenarios focusing coastal regions are not well documented, only a limited number of studies are available on the drinking water services at coastal schools. These studies focused on either RWH or tube wells (Islam et al., 2019; Rahman and Hashem, 2019) but none of the studies covered all existing technologies, both improved and unimproved, in the schools within the studied territory. Therefore, this literature left significant knowledge gaps, particularly addressing the overall status of water services in coastal schools. This study was conducted to understand the overall dynamics of drinking water supply infrastructure, water quality, and management in the coastal schools of Bangladesh. The findings will help decision-makers from local to the national level to take proper initiatives to ensure safe water supply in schools especially those located in the coastal area.

## 2 Methodology

### 2.1 Study site

Coastal areas in Bangladesh are highly vulnerable due to poor supply of water, while natural disasters like cyclone destroyed the majority of the water sources several times (Haque et al., 2010). Dacope Upazila has extensive problems with potable water because of lack of safe water sources, absence of sufficient infrastructure, inadequate financial allocation, and absence of proper relevant policies at the local level (Abedin and Shaw, 2013). Here, Abedin et al. (2014) observed that salinity is the main problem for the scarcity of fresh water in coastal areas, while the combined impacts of arsenic and drought along with salinity accelerated the health issues of coastal people. Also, skin diseases, dysentery, intestinal diseases, and diarrhea are very common waterborne diseases in these coastal areas.

**Figure 1:**
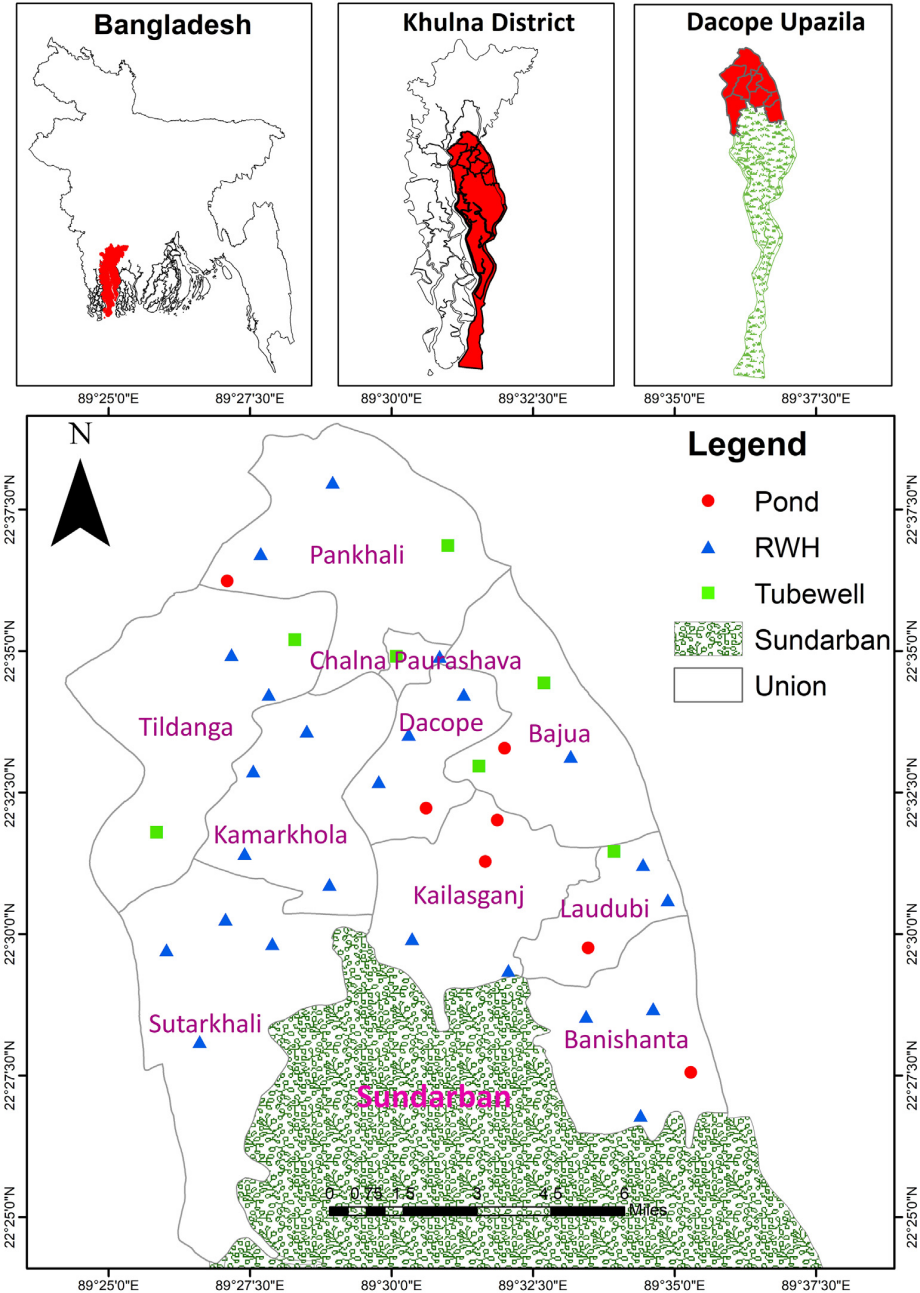
Map of the Study area indicating studied schools' location.

The study was carried out at Dacope Upazila (sub-district) of Khulna District in the southwestern coastal region of Bangladesh (Figure 1). The Upazila consists of 9 unions (Dacope, Bajua, Pankhali, Tildanga, Banishanta, Koilashganj, Loudubi, Sutarkhali, Kamarkhola) and one municipal town (Chalna) (BBS, 2012). As per (BBS (2012), there are 113 primary schools (56 government +57 registered) and 4 kindergartens in Dacope upazila. Nearly sixteen thousand students aged between 4 and 12 years are studying in these schools. About 450 teachers are employed in these schools. This study covered 38 government primary schools that represent 34% of the total schools where about 32% of the total students are associated.

### 2.2 Water sampling

Nearly 80% of the total rainfall in Bangladesh occurs between June to October. The quality of the harvested rainwater in this region tends to decline in time and being worst in the following pre-monsoon dry season (Chowdhury et al., 2020). Therefore, the water samples were collected during the pre-monsoon dry season (March–May, 2017) to address the extreme situation.

A reconnaissance field visit was conducted before the selection of the studied schools. This initial visit provided basic information about the schools of each union, particularly about the drinking water sources. School locations were recorded using a GPS machine (Garmin 62s, USA). Four schools were selected from each union following purposive sampling, which represents all available drinking water sources i. e. pond (*n* = 8), RWH (*n* = 24), and tube well (*n* = 6). Besides, two schools from the Chalna Municipality were studied. Therefore, a total of 38 (4 × 9 + 2) schools were selected for the present study (Figure 1).

A unique sample ID was assigned to each studied school (and their water sample) based on the name of their Union. One water sample from each school was collected following the grab sampling technique. Water samples were collected in sterile Nalgene plastic bottles. All the sampling bottles we labeled properly before sampling. Firstly, tube wells were pumped 20 times or outlet taps of harvested rainwater storage tank were run for at least 2 min. Then secondly, alcohol pads were used to disinfect the outlets. Finally, pre-sterilized and properly labeled Nalgene plastic bottles were used to collect the respective water samples. For pond, and RWH that do not have outlet tap, sampling bottles were rinsed three times with respective pond or RWH water and subsequently collected water through grab sampling.

There is only one pipeline that is connected to a single outlet and has no intermediate point and no multiple distribution systems. Therefore, we sampled water only from the single tap outlet. On the other hand, some schools manually withdraw water from the storage tank and has no outlet tap. In such case, we sampled water directly from the storage tank. Out of the 38 schools, 11 schools have in-house water filtration facility. Therefore, additional 11 water samples were taken from the point of use (filter outlet tap). Filter taps were also properly disinfected before collecting the sample. Hence, a total of 49 water samples were collected from the study area. Samples were stored in an insulated icebox (-4 °C) and transported to the laboratory for analysis within 12 h of sampling.

### 2.3 Key informant interview (KII)

A total of 38 KIIs were conducted with the head teacher (one teacher from each school with the presence of other teachers in the teacher’s common room) to understand the present scenarios of drinking water management in the schools. Questionnaires were finalized after conducting a pre-test during the reconnaissance survey in the study area. During this pre-test survey, teachers including a head teacher from randomly selected 5 schools were participated. Here, observations from preliminary field visit were included to prepare the final questionnaires. The interviews were conducted by the first two authors following the KII checklist (with a set of semi-structured open-ended questions) that includes drinking water source and its detailed physical characteristics, storage system, collection methods and uses, treatment facilities and the overall management (cleaning aspect, beneficiaries' group, using behavior, hygiene etc.) of the system. The responses are noted on a separate paper during the interview and immediately after the interview for analysis. Besides, a detailed physical inspection of each system in each site was done and noted by the first two authors to assess the condition of water supply infrastructures. Ethical consideration was maintained properly during this data collection. Written consent was taken before conducting KIIs and it was confirmed that all of the data will be presented in summative form, and all personal information will be maintained confidentially.

### 2.4 Laboratory analysis

A set of major water quality parameters from the Bangladesh drinking water quality standard, which are frequently used to study water quality in coastal Bangladesh were tested in the laboratory (DoE, 1997; Hossain et al., 2021; Rahman et al., 2017; Islam et al., 2011, 2019). Total coliforms (TC) and *Escherichia coli* bacteria were measured using membrane filtration technique (MFT) by following standard procedures (APHA, 2005) at the Environmental Microbiology Laboratory of the Environmental Science Discipline of Khulna University. A known volume of the sampled water (10 mL) was filtered aseptically into properly disinfected filtration assembly containing a sterile membrane filter (0.45 mm). Allowing a recovery period of 1 h, membranes were then incubated on Membrane Lauryl Sulphate Broth at 37 °C and 44 °C for *E. coli* and total coliforms respectively for 24 h. The standard plate count method was adopted to estimate the visible bacterial colonies. The colonies were expressed in terms of “colony forming units” (CFU) per 100 mL and calculated using Eq. (1):

(1) $$CFU/100\;mL\;of\;ample\;water=\;\frac{N*100}{DF}$$

Where, N = number of colonies counts and DF = dilution factor.

The pH, dissolved oxygen (DO) and salinity were measured on site using HACH Sension MM156 portable multi-parameter meter (HACH, USA). Chemical analyses including ammonia, nitrate, phosphate and sulfate were performed using a HACH DR 2700 Spectrophotometer following manufacturer protocols (Reagent Powder Pillows) at the Laboratory of the Department of Environmental Science and Technology, Jashore University of Science and Technology. Prior to all chemical analysis samples were filtered through 1.0 μm glass microfiber filters (Whatman® filter paper). Glass wares were thoroughly cleaned with 1% HNO_3_ (ACS reagent grade, Sigma-Aldrich) before every experiment. Use of double distilled water for standard preparation and sample dilution, reagent blanks and testing replication were adopted during analysis. Moreover, a random sub-set (n = 5) of the samples were tested in both laboratories to validate the results and found no significant inconsistencies.

### 2.5 Statistical analysis

Statistical analysis was performed using SPSS software (version 16.0). The Kruskal-Wallis H test was adopted to verify if there are statistically significant differences between the water sources on water quality parameters. Mann-Whitney U Test was performed to evaluate the water filtration efficiencies based on the Total Coliform and *E. coli* counts at the point of source and point of use. Figures and maps were produced with the help of Microsoft Office Excel (2016) and ArcGIS 10.3 version software respectively.

## 3 Results

### 3.1 Drinking water sources

**Figure 2:**
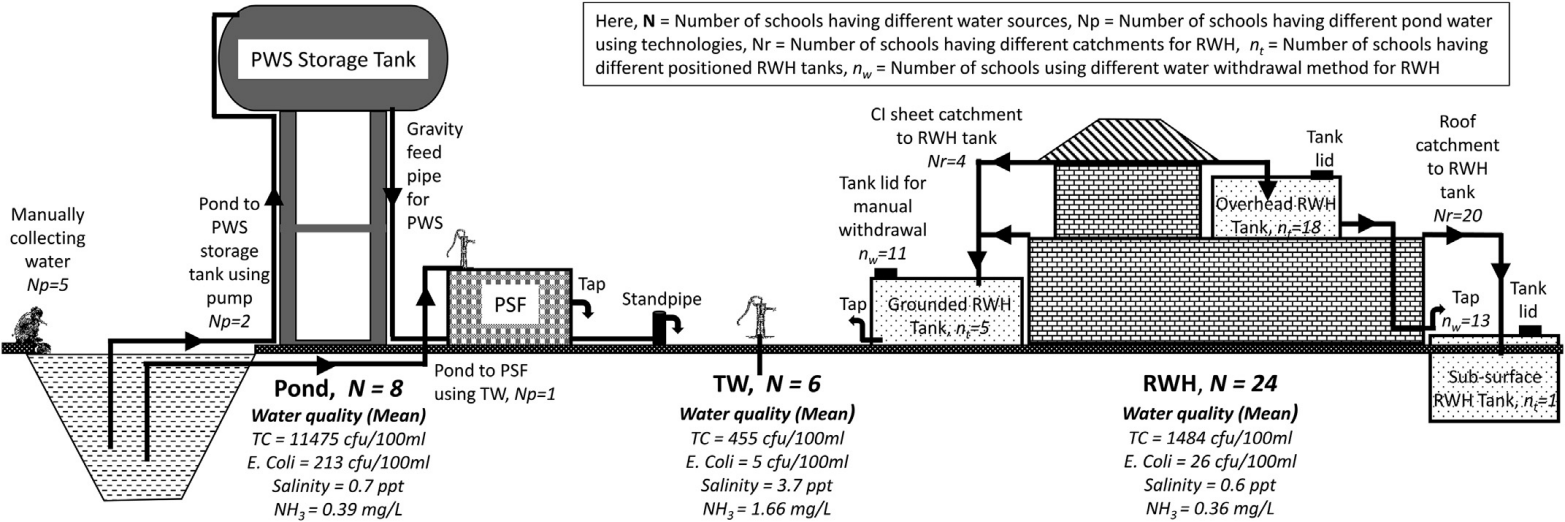
A schematic diagram of water supply systems found in studied school (Here, PWS = Piped Water Supply, PSF = Pond Sand Filter, TW = Tube Well, RWH = Rainwater Harvesting, TC = Total Coliform).

Among the studied schools, RWH (n = 24, 63%) was identified as the most common drinking water source followed by the rain-fed pond (n = 8, 21%) and tube wells (n = 6, 16%). The schools usually use the RWH system as the readymade water supply (without any treatment) during the rainy months (June–October) and store water for non-rainy months (November to May). Rain-fed Pond water-dependent schools withdraw pond water either directly (n = 5) or through pond sand filter (n = 1) and piped water supply (n = 2) (Source: KIIs). Figure 2 illustrates a detail schematic diagram of water supply systems found in the study area.

### 3.2 Physical characteristics of water supply systems

In RWH systems, a majority (n = 20, 83.33%) of the schools use concrete made roof as the catchment while four schools use corrugated iron (CI) sheet (Table 1). Polyvinyl Chloride (PVC) (n = 18, 75%) or concrete (n = 6) made tanks are used to store rainwater, having storage capacity over a range from 1,000 L to 20,000 L (mean = 10,277 L) and 4,000 L to 20,000 L (mean = 12,000 L), respectively and placed on overhead (n = 18), grounded (n = 5) and subsurface (n = 1). Half of the schools have adopted manually inserting bucket method for water collection, while the rest have installed outlet water taps. Only 25% of the RWH dependent schools had an in-house mineral pot filter-based water treatment facility. All the RWH systems were owned by the schools and use manual first flushing systems for maintenance purposes. The flushing frequencies ranged between 1 to 10 times in every five years. The managing committee of the schools (a group of stakeholders, usually selected by the local administration, empowered for overall management supports) performs the responsibility for the operation and maintenance of the RWH system. However, there were no water quality monitoring program and poor maintenance were observed at most of the RWH systems during the inspection (Source: KIIs and onsite observation).

**Table 1:** Characteristics of RWH systems in the studied schools.

Sample ID	Roof material	Roof age (years)	Water route	Tank material	Position of tank	Storage capacity (L)	Tank age (years)	Treatment before tank storage	First flushing	Collection medium	Treatment before consumption
D2	Concrete	20	R-G-D-T	Plastic	Overhead	15000	7	No	MFF	Tap	No
D3	Concrete	40	R-D-T	Plastic	Overhead	1000	5	No	MFF	Tap	MPF
D4	Concrete	17	R-D-T	Plastic	Ground	1000	10	CF	MFF	Manually	MPF
B4	Concrete	11	R-G-D-F-T	Plastic	Overhead	15000	4	SGBCF	MFF	Tap	No
P2	Concrete	14	R-D-F-T	Plastic	Overhead	16000	2	SGBCF	MFF	Tap	No
P3	Concrete	12	R-G-D-F-T	Plastic	Overhead	15000	2	SGBCF	MFF	Tap	No
T2	Concrete	15	R-G-D-T	Plastic	Overhead	4000	3	No	MFF	Manually	No
T4	CI sheet	7	R-G-T	Plastic	Overhead	15000	5	No	MFF	Manually	No
BS1	Concrete	20	R-D-T	Concrete	Sub-surface	15000	16	No	MFF	Manually	MPF
BS3	Concrete	8	R-G-D-F-T	Concrete	Ground	20000	2	SGBCF	MFF	Tap	MPF
BS4	Concrete	5	R-D-T	Concrete	Overhead	3000	5	No	MFF	Tap	No
K1	Concrete	20	R-D-F-T	Plastic	Overhead	15000	3	SGBCF	MFF	Tap	No
K2	Concrete	6	R-D-T	Concrete	Ground	5000	6	No	MFF	Manually	MPF
L3	Concrete	8	R-D-F-T	Plastic	Overhead	15000	8	SGBCF	MFF	Tap	MPF
L4	CI sheet	12	R-G-D-T	Concrete	Ground	8000	8	No	MFF	Manually	No
C1	Concrete	6	R-D-T	Plastic	Overhead	1000	4	No	MFF	Manually	No
C2	Concrete	14	R-G-D-T	Plastic	Overhead	8000	3	No	MFF	Manually	No
S2	Concrete	8	R-D-T	Plastic	Overhead	1000	8	No	MFF	Manually	No
S3	Concrete	4	R-D-T	Plastic	Overhead	8000	4	No	MFF	Tap	No
S4	CI sheet	7	R-G-D-T	Plastic	Overhead	2000	5	No	MFF	Manually	No
KA1	CI sheet	18	R-G-D-T	Plastic	Overhead	2000	7	No	MFF	Manually	No
KA2	Concrete	22	R-D-T	Concrete	Overhead	5000	9	No	MFF	Tap	No
KA3	Concrete	14	R-D-F-T	Plastic	Overhead	20000	8	SGBCF	MFF	Tap	No
KA4	Concrete	8	R-D-F-T	Plastic	Ground	20000	2	SGBCF	MFF	Tap	No

CI = Corrugated Iron, R = Roof, G = Gutter, D = Downpipe, F = Filtration, T = Tank, CF = Cloth filtration, SGBCF = Sand gravel brick chips filtration, MFF = Manual First Flushing, MPF = Mineral Pot Filter^1^. [Prepared on the basis of KII].

Except for a deep tube well (B2), all the other schools have shallow hand-operated tube wells (Table 2). All the tube wells owned by the schools and located within the school premises. Besides, out of 6 schools, 5 of them collect tube well water manually and consume directly without any purification. Only one school (B3) used to filter (mineral pot filter) before consumption (Source: KIIs and onsite observation).

**Table 2:** Characteristics of Tube wells in the selected schools.

School ID	Source owner	Type of TW	Collection medium	Treatment before consumption	Responsibility of O&M
B2	School	DTW	Manually	No	School committee
B3	School	STW	Manually	MPF	School committee
P4	School	STW	Manually	No	School committee
T1	School	STW	Manually	No	School committee
T3	School	STW	Manually	No	School committee
L2	School	STW	Manually	No	School committee

DTW = Deep Tube Well, STW = Shallow Tube Well, MPF = Mineral Pot Filter. [Prepared on the basis of KII].

Among the pond water using schools, half of them (n = 4) have their pond within the school premises and the rest of the ponds are private except a community-owned pond (Table 3). Out of eight ponds, three ponds had no protection to protect surface runoff and cattle disturbance. Two ponds are protected by high dike and fence all around, and two ponds have dikes without fence. Five schools collect pond water manually using pitcher, two schools lift (using electric motor) water into a storage tank and thereafter use gravity flow piped water supply (PWS) and the rest uses hand operated tube well to feed the pond sand filter (PSF). The responsibility of operation and maintenance of ponds and supply medium rely on the respective committee or the owner of the system. Water treatment facilities were available only in half of the pond water using schools using mainly mineral pot filter (n = 3) and alum treatment in one school (B1) (Source: KIIs and onsite observation).

**Table 3:** Characteristics of ponds in the selected schools.

School ID	Source owner	Collection medium	Secondary Storage	Treatment before consumption	Source protection	Responsibility of O&M
D1	Govt.	Manually	Plastic tank	MPF	Dyke	Community
B1	Private	Manually	No	Alum	Fence & dike	Owner
P1	Private	Manually	No	MPF	No	Owner
BS2	Govt.	Manually	No	MPF	No	Local Govt.
K3	Govt.	PWS	No	No	Fence & dike	NGO
K4	Govt.	Manually	Plastic bucket	No	No	School committee
L1	Community	PWS	No	No	Dyke	Community
S1	Private	PSF	No	No	No	PSF committee

PWS = Piped Water Supply, PSF = Pond Sand Filter, MPF = Mineral Pot Filter [Prepared on the basis of KII].

### 3.3 Water quality

Physicochemical and biological characteristics of studied RWH, tube well and pond water at point of source along with their comparison with Bangladesh standards (DoE 1997) for drinking water are represented in Table 4. A Kruskal-Wallis H test revealed that there was a statistically significant differences in pH, DO, salinity, NH_3_, SO_4_, PO_4_ and TC among the drinking water sources (all *p*-values < 0.05). While NO_3_ and *E. coli* do not show statistically significant differences among the sources at 5% confidence interval. The pH of RWH (mean 7.8) is significantly higher as compared those of pond (mean 7.5) and tube well (mean 7.5) waters. In contrast, DO of RWH (mean 9.2 mg/L) shows statistically significant lower concentrations than those of tube well (mean 11.2 mg/L) and pond water (mean 10.5 mg/L). Three of the water samples (two RWH and one pond) showed non-conformance of DO standard for Bangladesh (6 mg/L). Tube well water exhibited significantly higher salinity (mean 3.7 ppt) as compared those of RWH and pond. Tube wells and one-fourth of the pond water failed to qualify against salinity standard for Bangladesh (1 ppt).

**Table 4:** Water quality of drinking water sources in the selected schools of Dacope Upazila

Parameter	Unit	BDS	Mean concentration (% of non-conformity)			Chi-square (χ2)	*df*	*P*-value
			RWH	TW	Pond			
pH	N/A	6.5–8.5	7.82 (4.17)	7.54 (16.67)	7.55 (000)	9.191	2	0.010∗
DO	mg/L	6	9.28 (8.33)	11.20 (000)	10.51 (16.67)	8.188	2	0.017∗
Salinity	Ppt	1	0.06 (000)	3.72 (100)	0.71 (25.00)	19.505	2	0.000∗∗∗
NH_3_	mg/L	0.5	0.36 (12.50)	1.66 (83.33)	0.40 (37.50)	6.510	2	0.039∗
NO_3_	mg/L	10	0.78 (000)	2.57 (000)	1.64 (000)	2.711	2	0.258
SO_4_	mg/L	400	5.63 (000)	0.00 (000)	36.88 (000)	13.781	2	0.001∗∗
PO_4_	mg/L	6	0.10 (000)	0.42 (000)	0.21 (000)	7.537	2	0.023∗
TC	cfu/100 mL	0	1484 (100)	455 (100)	11475 (100)	7.467	2	0.024∗
*E. coli*	cfu/100 mL	0	26 (58.33)	5 (16.67)	213 (75.00)	5.025	2	0.081

DO = Dissolved Oxygen, TC = Total Coliform, *E. coli = Escherichia coli,* TW = tube well, RWH = Rainwater Harvesting, BDS = Bangladesh standard, df = degrees of freedom. Here, ∗∗∗P < .001; ∗∗P < .01; ∗P < .05.

The average concentration of ammonia and nitrate in tube well water (NH_3_ = 1.66 mg/L, NO_3_ = 2.56 mg/L) was higher than RWH (NH_3_ = 0.36 mg/L, NO_3_ = 0.77 mg/L) and pond water (NH_3_ = 0.39 mg/L, NO_3_ = 1.6 mg/L), respectively. However, the differences are not statistically significant at 5% (χ2 (2) = 2.711, *p* = 0.258). Except for a tube well of one school (T3), all samples had exceeded the Bangladesh limit for ammonia 0.5 mg/L. On the other hand, sulfate was not detected in any tube well water but found high concentrations in RWH (mean = 5.6 mg/L) and pond (mean = 36.8 mg/L) water. Phosphate concentration was higher in tube well samples than that of the RWH and pond water. The highest value of nitrate and phosphate found in the pond water of a school (P1), a private pond that was reported as a fish cultivation pond where fish feed was frequently used. Overall, sulfate and phosphate in all samples were within the Bangladesh standard.

Microbial contamination (both total coliform and *E. coli*) was much higher in the pond water than that of the RWH and tube well water. Total coliforms (TC) were detected in all samples over the Bangladesh standard (0 cfu/100 mL) but the highest concentration was observed in pond water. The *E. coli* was found in two-thirds of RWH, three-fourths of pond water and one tube well samples where the standard is also 0 cfu/100 mL. The median value of *E. coli* found in rainwater was 8 cfu/100 mL, ranging from 0 – 210 cfu/100 mL while the median value in pond water was 135 cfu/100 mL, ranging from 0 – 600 cfu/100 mL. In RWH, both highest *E. coli* and TC were found in the concrete made storage tank, where concrete roof catchment was found very muddy with excessive growth of algae and shrubs. Moreover, the faulty construction of the RWH tank’s lid allows runoff to enter into the tank during the rainy season. In pond water, the highest concentrations of *E. coli* and TC were found in different ponds. Those schools have several cricks to allow surface runoff into the pond. Besides, several cowsheds were found in the periphery and ducks were floating often in the pond (Source: KIIs and onsite observation).

### 3.4 Efficiency of in-house water treatment for bacterial removal

The efficiency of in-house water treatment was measured by comparing the *E. coli* concentration of source and treated water. Treatment of drinking water before consumption was found only in 26% of the schools (Supplementary Figure 1). All filtration systems (MPFs) were installed in the teacher’s common rooms. However, access to the filtered water was limited to the teachers only except in one primary school where both teachers and students drink pond water treated by Alum. Surprisingly, total coliform (TC) in 5 schools out of 11 was recorded higher in point of use water than the source water (Supplementary Figure 2a). Similarly, among those five schools, two schools have also evaluated increased *E. coli* concentration after the filtration as compared than that of source water (Supplementary Figure 2b). Daschner et al. (1996) also found higher bacterial count in filtered water than the source water due to long storage and irregular cleaning of the filter. We also found long storage time (7–30 days) and irregular cleaning of the filters during field investigation. In rest of the schools, the efficiency of MPF in reducing TC and *E. coli* was in a range of 40–98% and 84–100%, respectively. Alum treatment in one school reduced 96% TC and 100% *E. coli*. However, a Mann-Whitney U test suggests that the increase or decrease of TC and *E. coli* count in point of use water are not statistically significant different than that of source water at 5% (*U* = 36.5, *p* = 0.307 for TC and *U* = 29, *p* = 0.09 for *E. coli*).

### 3.5 Health risk of indicator bacteria

WHO (1997) risk category was used to classify the risk of total *E. coli* counts found in different water sources and illustrated in Table 5. Treated *E. coli* concentration of that school, where both teachers and students drink treated water, was included in health risk assessment while the treatment results of other schools were excluded since the facility was dedicated to teachers only. The majority of the tube wells water had no risk. Also, almost half of pond (37.5%, n = 3) were at high risk and 37.5% RWH (n = 9) was within the intermediate risk. Overall, 44.7% of samples fall in no risk, 10.5% in low risk, 31.6% in intermediate-risk and the rest 13.2% in the high-risk category. The spatial distribution of risk classes over Dacope Upazila was shown in Supplementary Figure 3.

**Table 5:** Health risk category of drinking water sources.

Risk category	Range of *E. coli* (cfu/100 mL)	RWH		TW		Pond		Total	
		n	%	n	%	n	%	N	%
No	<1	9	37.5	5	83.3	3	37.5	17	44.7
Low	1–10	4	16.7	0	0	0	0	4	10.5
Intermediate	>10–100	9	37.5	1	16.7	2	25	12	31.6
High	>100 - 1000	2	8.3	0	0	3	37.5	5	13.2

*E. coli = Escherichia coli,* RWH = Rainwater Harvesting, TW = Tube Well.

## 4 Discussion

School requires sufficient supply of safe drinking water because school-going children used to spend most of the daytime in school. Moreover, they are more vulnerable to different kinds of water-borne diseases e.g., diarrhea, jaundice, cholera, etc. Such occurrences are more common in developing countries and water-stressed areas like Bangladesh. Among the coastal areas in the southern part of Bangladesh, Dacope Upazilas is one of the most drinking water-stressed areas where school-going children are struggling to have sufficient water. This study focused on the present conditions of schools in the perspective of drinking water supply infrastructure, quality, and management.

The present study shows that the schools in the coastal area use different types of drinking water sources such as RWH, tube well, pond. In all the studied tube wells, salinity was extremely exceeded the threshold value for Bangladesh (Table 4). Many of the previous studies in this region also identified higher groundwater salinity in both shallow and deep aquifer (Rakib et al., 2020; Zahid et al., 2018). Some of the ponds water also detected for slightly higher salinity which might be due to the influence of tidal water and the interaction with a highly saline shallow aquifer (Worland et al., 2015). This high saline contamination of drinking water sources might be the cause of higher rates of (pre)eclampsia and gestational hypertension of pregnant women in coastal Bangladesh (Khan et al., 2014). The problem may be exacerbated by future sea-level rise and environmental change, while people are experiencing emerging health issues nowadays (Chowdhury et al., 2020; Khan et al., 2011). Besides, Akter (2019) reported that exposure to saline drinking water decreases children's grade advancement in coastal Bangladesh. In the present study, salinity was not detected in RWH samples and consistent with the previous results (Islam et al., 2019). Therefore, schools either dependent on tube well or pond for drinking water supply are planning to move towards a safer option through the aid of donor agencies or public investment. However, those opportunities are limited due to financial strength and technical incapability to implement and maintenance (Source: KIIs). Also, Abedin et al. (2014) summarized that the majority of the schools had to utilize rainwater as a source of drinking water mainly due to the increasing rate of salinity in coastal aquifers of Bangladesh.

Ammonia level is lower than Bangladesh standard (DoE, 1997) in harvested rainwater (except sample no. BS4, S2, S3, and KA4) whereas comparatively higher in tube well water (5 out of 6 samples). Besides, among the eight pond water sources, three sources contain ammonia above the Bangladesh standard (0.5 mg/L). The influence of septic tank and other anthropogenic and natural disturbance may increase the ammonia concentration in surface and shallow groundwater (Du et al., 2017), which was consistent with the current field investigation. However, the concentration of nitrate, sulfate, and phosphate found in all of the collected sample water below the described standard by Bangladesh.

Contamination of microbial pathogens was found in all types of water sources (Table 4), which was expected since previous studies on drinking water quality in this region revealed high bacteriological contamination in water sources (Islam et al., 2011, 2019). However, microbial contamination of tube well water is rare. In this study, all tube well water is contaminated by total coliforms. Besides, the shallow tube well water of one of the schools (L2) showed the presence *E. coli* which surely indicated the contamination by human or animal feces. One possible mode of contamination would be when someone touched the outlet of the tube well after defecation without maintaining proper hygiene and bacteria attached to the rough surface of outlet and grow quickly (Mahmud et al., 2019). Moreover, all the tube wells were installed in an open place without rooftop so that contamination of total coliform, even *E. coli* can come from the human disturbance and most often by bird’s excreta.

Pond water is naturally more susceptible to microbial contamination. Besides, Islam et al. (2011) also found higher bacterial count in drinking water pond in coastal Bangladesh. During field investigation, the majority of the ponds were found unprotected from surface runoff, cattle disturbance and unhygienic practices i.e. bathing, washing which could contaminate pond water. Though total coliform was detected in all the pond water, no *E. coli* was found in two ponds out of eight ponds both have high dike and fence and not managed by the school managing committee like the other ponds. Moreover, one of them was managed excellently by its owner and the other use a pond sand filter to purify pond water before supply. This indicates proper management is one of the key factors to obtain safe drinking water.

Rainwater Harvesting (RWH), the major source of drinking water at primary schools in Dacope Upazila was found to be contaminated by coliforms. Our physical inspection during sampling revealed that the several possible causes of contamination that occurred in harvested rainwater. Firstly, the muddy and dirty roof catchment where large shrubs, algae and fungi have already grown up, which indicated poor or no maintained at all. Secondly, all the school uses manual first flushing which was found in bad conditions and almost unusable. Thirdly, irregular cleaning of rainwater harvesting tanks resulted in a huge amount of settled pollutants at the bottom of the tank that also indicated the poor use of the first flushing system. Fourthly, lack of proper maintenance of hygiene and safety during water handling. Almost half of the schools practice manual water collection using a pot which was uncleaned and increase the risk of contamination. Most of the cases, the collection pot which was inserted to uptake water from the storage tank itself used as a secondary storage jar at the consumption point that was being placed on a dirty surface. Consequently, students handled this jar for drinking water and again inserted it into the storage tank without cleaning properly. As a result, the tank water is getting contaminated day by day which might be a big risk factor for increasing bacterial pathogens in tank water. Fifthly, some construction errors such as faulty construction of tank lid and roof allow polluted water to enter the tank during rainfall. Also, we found that a harvested rainwater tank is situated in the subsurface where heavy rainfall can submerge the entrance of the tank and seepage can occur. Finally, irregular monitoring of the entire system was found due to a lack of resources and staff. Moreover, the staffs who are responsible for water handling in school were untrained. Consequently, they have limited or no idea on how to maintain the cleanliness of the system, leading to bacteriological contaminations. Previous studies also found some of those findings on bacterial contamination of harvested rainwater (Islam et al., 2011, 2013, 2019).

Furthermore, the majority of schools do not use any type of treatment process before drinking. Those schools are using the treatment process, in most of the cases, students have no access. This is occurred due to the lack of facilities and resources in school as the available treatment devices are neither funded from the government allocation nor the school’s fund. Teachers used to install these devices personally for their safety and keep the filter unit or treatment facility in their staff room while students used to drink untreated source water. Even though using mineral pot filter (MPF) to treat source water, bacterial contamination was found in treated water. It happens most probably due to irregular cleaning of MPF, long term water storage in MPF, unsafe water handling during watering and most importantly, not changing the filtration device that has to change periodically for efficient performance. Also, Karim et al. (2016) assessed the performance of MPF in reducing coliforms from drinking water which showed inconsistency in removal efficiency. On the other hand, alum treatment showed very high efficiency in bacterial removal, nevertheless, it cannot be concluded based upon a single observation.

Majority of the schools' water are at high health risk according to WHO (1997) risk category. Tube wells water had the lowest risk but unusable due to high salinity. Besides, pond water was at higher risk with highest microbial contamination. Comparatively, RWH can provide water with lower risk, but proper maintenance and disinfection is highly demanded. Highlighting the importance of removing *E. coli* regarding child health, Black et al. (1982) stated that *E. coli* is highly related to the diarrheal diseases of the child in Bangladesh. Beside, Sobsey et al. (2003) discussed that proper treatment is mandatory to ensure safe drinking water and this is related to education, awareness, and attitude related aspects of the people to ensure hygiene. Since the high concentration of TC and *E. coli* are responsible for various forms of health risk, urgent attention is required for school-going children as they are very susceptible to waterborne disease.

## 5 Conclusion

This research analyzed the overall situation related to drinking water aspects e.g. supply infrastructure, quality and management in primary schools of Dacope Upazila in coastal Bangladesh. Among the three types of drinking water sources e.g. ponds, tube wells, RWH systems, a considerable number of vital water quality parameters found above the standard level of Bangladesh. Contamination of harmful bacteria is present in all types of drinking water sources, even in treated water. The harvested rainwater had not been stored in an appropriate structure with proper cleanliness. The tube well water was highly saline and far behind to reach the acceptable limit of Bangladesh. Though ponds require most intensive maintenance, it was found as the most neglected source and provide the worst quality of water. Overall, RWH is a better source in the study area, but lack of resources restricts other schools to shift into RWH.

Majority of the sources has high health risk. At this stage, however, we are currently endangering a large number of children who will hold the flag of development in the long run for this vulnerable coastal community. Against the backdrop presented above, it can be concluded that RWH could be a safer option for the schools in coastal Bangladesh if disinfected well before consumption. Therefore, adopting a safe and effective disinfection to remove microbial pathogens is an urgent need. Besides, proper construction of the RWH system with stronger maintenance strategies along with providing sufficient resources to look after the water services in schools should be ensured. In this case, the governing authority of schools could take initiatives like deploying a specific person who will be responsible for cleaning and maintaining the water services. Alternatively, teachers could perceive their problem and could ensure the three most important measures for potability i.e., (i) source protection, (ii) point of use water treatment and (iii) regular cleaning and maintenance of water technologies and source.

Additionally, government and non-government agencies may initiate suitable water supply projects with adequate funding to supply safe drinking water at schools. Besides, adopting a water safety plan along with training and awareness-raising programs should be run to make people aware of the safe management of drinking water. Additionally, research institute should come across to develop affordable and easily operable in-house water treatment devices to ensure safe drinking water. Further study can be conducted to understand the public health issues regarding water quality in coastal schools of Bangladesh, where governance analysis maybe done to understand the possibility and constraints to ensure sustainable safe water management.

This study was conducted before the COVID-19 pandemic in 2017. The situation may have changed since then with the continuous progress of the drinking water supply in Bangladesh. Due to resource constraints, we were unable to study the progress and assess the current water supply scenario in the primary schools in coastal Bangladesh. However, the findings in this study would be helpful for the decision-maker to plan and implement water supply projects in coastal Bangladesh and similar settings.

## Declarations

### Author contribution statement

Mohammad Jobayer Hossain: Conceived and designed the experiments; Wrote the paper.

Md. Ansarul Islam: Performed the experiments; Analyzed and interpreted the data; Wrote the paper.

Md. Hasibur Rahaman: Conceived and designed the experiments; Analyzed and interpreted the data; Wrote the paper.

Md. Arif Chowdhury: Analyzed and interpreted the data; Wrote the paper.

Md. Atikul Islam: Conceived and designed the experiments; Performed the experiments; Contributed reagents, materials, analysis tools or data.

Mohammad Mahfuzur Rahman: Conceived and designed the experiments; Analyzed and interpreted the data; Contributed reagents, materials, analysis tools or data.

### Funding statement

This research did not receive any specific grant from funding agencies in the public, commercial, or not-for-profit sectors.

### Data availability statement

Data will be made available on request.

### Declaration of interests statement

The authors declare no conflict of interest.

### Additional information

No additional information is available for this paper.

## Footnotes

1 MPFs are portable water treatment device commercially produced and widely used in Bangladesh and other south Asian countries.
