# Supplementary material for: Drinking water services in the primary schools: evidence from coastal areas in Bangladesh
Source: Heliyon. 2022 Jun 23;8(6):e09786. doi: 10.1016/j.heliyon.2022.e09786 (PMC9241045; doi:10.1016/j.heliyon.2022.e09786)
Supplement: Supplementary File [file mmc1.docx]

**Key Informant Interview Checklist**

**Case Study on water supply in the primary schools at Dacope Upazila Project**

**Position of the Key Informant in the school:**

1. Head teacher

2. Assistant teacher (if head teacher is absent)

**Consent form**

As-salamu alaikum. I am a final year bachelor student at the Department of Environmental Science and Technology, Jessore University of Science and Technology. I am collecting drinking water samples from the primary schools from your locality and some relevant information for academic purpose. The aim of this study is to evaluate the quality of the drinking water and associated health risks. I would like to know some information regarding the water source, storage systems, maintenance and use. All the information will be used for academic purpose only. Would you please agree to cooperate with us?

**KII Checklist:**

1. **Background information of the school**

- Name
- Number of teacher and students
- Address

1. **Description of the drinking water system**

- Main source/technology of water and ownership

What is the main source/technology of water for this school? Who owns this source/technology?

- Location of the source/technology, protection of the source/technology

Where is the source/technology located? What kind of protection measures are taken? Anything important you would like to mention for this source/technology’s location and protection?

- Detailed physical characteristics of the source/technology

How many years this source is being used? What is the capacity of this source? Which is the construction material? Anything important you would like to mention for this source/technology’s physical characteristics?

- Storage system, collection methods and uses

How is the water stored? If any tank is used, Which the material for water storage tank? How do you collect water? What are the main uses? Anything important you would like to mention for this source/technology’s storage system, collection methods and uses?

- Treatment facilities, method and beneficiaries

Is there any treatment facility? If yes, what is that? When does it occur? Do you use any in-house treatment facility? If yes, how do you do that? Who uses this system? Anything important you would like to mention for this source/technology’s treatment facilities?

- Responsible management authority and maintenance frequency

Who is mainly responsible for O&M? How frequently regular maintenance are done? If any emergency maintenance required, who do that? Who they fund the O&M? Anything important you would like to mention for this source/technology’s O&M?

**Detailed physical investigation:**

- Current physical state of source/technology and its protection
- Surrounding environment and position
- Water route/distribution system and collection method
- Secondary storage/treatment system

**Supplementary Data**

**Supplementary Figure 1: Percentage of in-house water treatment user, using methods and its beneficiaries**

**
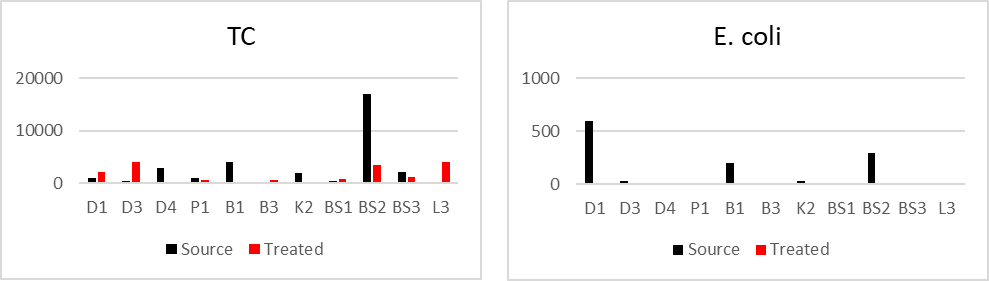
**

1. **(b)**

**Supplementary Figure 2: Efficiency of water treatment in bacterial removal**

**
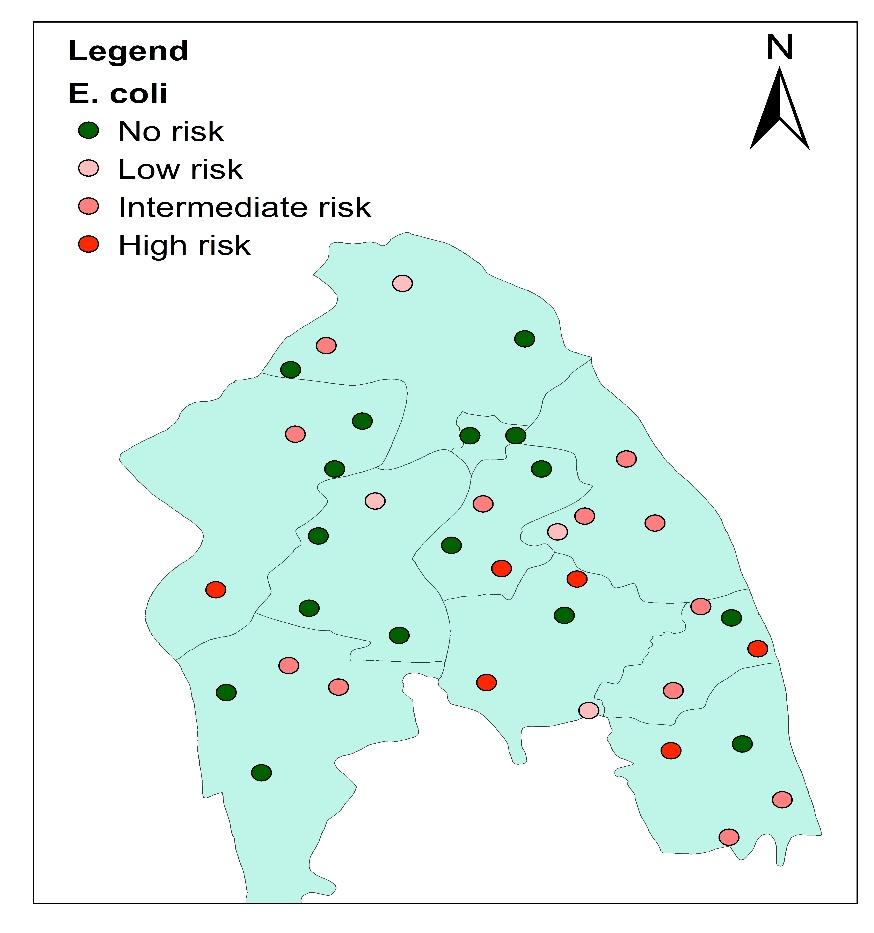
**

**Supplementary Figure 3: Risk category of drinking water sources**
